# The Input of Nanoclays to the Synergistic Flammability Reduction in Flexible Foamed Polyurethane/Ground Tire Rubber Composites

**DOI:** 10.3390/ma17215344

**Published:** 2024-10-31

**Authors:** Aleksander Hejna, Paulina Kosmela, Adam Olszewski, Wiktoria Żukowska

**Affiliations:** 1 Institute of Materials Technology, Poznan University of Technology, 3 Piotrowo St., 61-138 Poznan, Poland; 2 Department of Polymer Technology, Gdansk University of Technology, 11/12 Narutowicza St., 80-233 Gdansk, Poland; paulina.kosmela@pg.edu.pl (P.K.); adam.olszewski@pg.edu.pl (A.O.); wiktoria.zukowska@pg.edu.pl (W.Ż.)

**Keywords:** polyurethane foams, ground tire rubber, composites, flammability, flame retardants

## Abstract

Currently, postulated trends and law regulations tend to direct polymer technology toward sustainability and environmentally friendly solutions. These approaches are expressed by keeping materials in a loop aimed at the circular economy and by reducing the environmental burdens related to the production and use of polymers and polymer-based materials. The application of recycled or waste-based materials often deals efficiently with the first issue but at the expense of the final products’ performance, which requires various additives, often synthetic and petroleum-based, with limited sustainability. Therefore, a significant portion of research is often required to address the drawbacks induced by the application of secondary raw materials. Herein, the presented study aimed to investigate the fire performance of polymer composites containing highly flammable matrix polyurethane (PU) foam and filler ground tire rubber (GTR) originating from car tire recycling. Due to the nature of both phases and potential applications in the construction and building or automotive sectors, the flammability of these composites should be reduced. Nevertheless, this issue has hardly been analyzed in literature and dominantly in our previous works. Herein, the presented work provided the next step and investigated the input of nanoclays to the synergistic flammability reduction in flexible, foamed PU/GTR composites. Hybrid compositions of organophosphorus FRs with expandable graphite (EG) in varying proportions and with the addition of surface-modified nanoclays were examined. Changes in the parameters obtained during cone calorimeter tests were determined, discussed, and evaluated with the fire performance index and flame retardancy index, two parameters whose goal is to quantify the overall fire performance of polymer-based materials.

## 1. Introduction

Economic development permanently stimulates the development of the plastics market, including the branch of polymeric foams. According to different market reports, the global polymer foam market is valued at ~USD 125 billion [1,2,3] after partial recovery from the COVID-19 pandemic and the military conflict in Ukraine. The forecasts indicate its future growth at a compound annual growth rate above 5% by 2032 [4]. The increasing demand for lightweight and versatile materials in packaging, automotive, furniture and bedding, construction, aerospace, and other industries drives the polymer foam market. Along with the industrial revolution, the scientific environment also follows current trends and aims to develop novel material and process solutions related to polymeric foams.

Among their most common types are polyurethane (PU) foams, whose popularity is attributed to their excellent performance and broad range of potential innovations, which also stimulate related research activities. Keeping in mind the applications of PU foams, current sustainability-driven trends and regulations noticeably affect their development. On the one side, they are related to the incorporation of renewable or recycled resources as raw materials for PU foams [5,6,7,8,9,10], but on the other one, they require minimizing foams’ environmental burdens, which are attributed to their flammability [11,12,13]. Most of the polymers without proper modifications are conventionally flammable [14,15]. To address this drawback, flame retardants (FRs) are applied, the goal of which is to enhance the stability of the material and limit its degradation when subjected to fire. However, due to the multifaceted threats posed by the fire, these additives need to address multiple issues. Proper selection and adjustment of FRs and their compositions enhancing the inherent efficiency of protective layer formation should be the priority during PUFs’ modifications aimed at flammability reduction, along with the beneficial activity in the gas phase.

Currently, the market offers a multitude of potential FRs differing in type, chemical structure, and mode of action. One of the most widely used classifications is their division into additive and reactive, which is associated with their interactions with polymer material [16,17]. Additive FRs are applied similarly to fillers, as they are hardly bonded to the polymer macromolecules, typically mechanically quenched or bound through electrostatic or hydrogen interactions [18]. Such a nature of interfacial interactions yields potential migration from polymer materials, which has been repeatedly analyzed as a potential threat [19,20]. However, the application of additive FRs is very straightforward, as it typically includes only additional components during material preparation.

On the other hand, reactive FRs are typically incorporated during synthesis or polymerization as additives or even monomers [21,22]. It yields their strong, typically covalent bonding with the macromolecular structure, significantly inhibiting their migration during product use and limiting health and environmental threats. However, there are always two sides to the same coin, and in the case of reactive FRs, the limitations are often related to the application of more complex manufacturing procedures, either considering FRs’ production or their incorporation into polymer materials.

The other FR divisions, based on their chemical structures, were repeatedly reviewed in multiple papers [23,24,25,26]. Due to the multitude of FRs offered by the market, multiple classifications could be developed, but considering PU foams, the most common groups are phosphorus, nitrogen compounds, expandable graphite (EG), inorganic FRs, and nanofillers. The nitrogen-based FRs are mainly related to the melamine and its derivatives, which inhibit flammability by the absorption of heat and the generation of non-combustible nitrogen-based gases resulting from thermal decomposition, mainly ammonia [27]. Phosphorus-based FRs include inorganic and organic compounds characterized by the reduced release of toxic combustion products, reduced smoke emission, and increased flame retardancy [28,29]. They typically provide a vital contribution to the protective char layer formation, which limits the heat and mass exchange with the environment, hindering further combustion [30,31]. They are often considered the most efficient FRs for PU foams, and noticeably, their efficiency can be significantly boosted by the combination with nitrogen-based additives. A very auspicious and commonly applied FR for PU foams is EG, whose mechanism is based on the enormous volumetric expansion and generation of a carbonaceous layer, which protects the material’s surface. The protective effect is based on the limited heat and mass transfers, which inhibit the fire spread [32,33]. Similar to previous FR groups, it can be effectively applied as a component of more complex FR systems [34,35]. Inorganic FRs are typically based on the endothermic dehydration, which dilutes the volatile products of thermal decomposition and reduces toxic smoke emission. However, they typically require high concentrations (often over 50%), which significantly contribute to the deterioration of the mechanical performance [36]. Finally, nanofillers offer exciting opportunity to deal with the greatest disadvantage of inorganic FRs. Due to their particle size and related surface area, they require significantly lower loadings to provide efficient flame retardancy to polymer materials [37,38,39].

In the case of PU foams and other cellular materials, there is also another vital division related to the FR application method. The first one involves the introduction during polymerization or foaming, indicating that they are embedded in the whole volume of the material. The second one is related to the surface coating of foams, which assumes the modification of previously prepared material. These approaches noticeably differ and require various considerations regarding the performance of applied FRs [40,41].

Considering the aforementioned aspects and the well-known sensitivity of PU foams to changes in formulations and the introduction of additives, the application of FRs should simultaneously address the ignitability, high flammability, and smoke generation of foams without the simultaneous deterioration of structure and performance. Such deterioration can be expressed by the plasticizing effects of organophosphorus FRs [42,43,44] or the enhanced friability of cellular structure resulting from the incorporation of solid FRs, e.g., melamine and its derivatives [45,46,47]. It has been repeatedly reported that single-component approaches are limited in action [48,49]. To efficiently address the foams’ flammability and hindered performance deterioration, the combination of FRs with different modes of action should be applied, which balances the flammability-related benefits with the quality of a cellular structure and performance.

**Figure 1:**
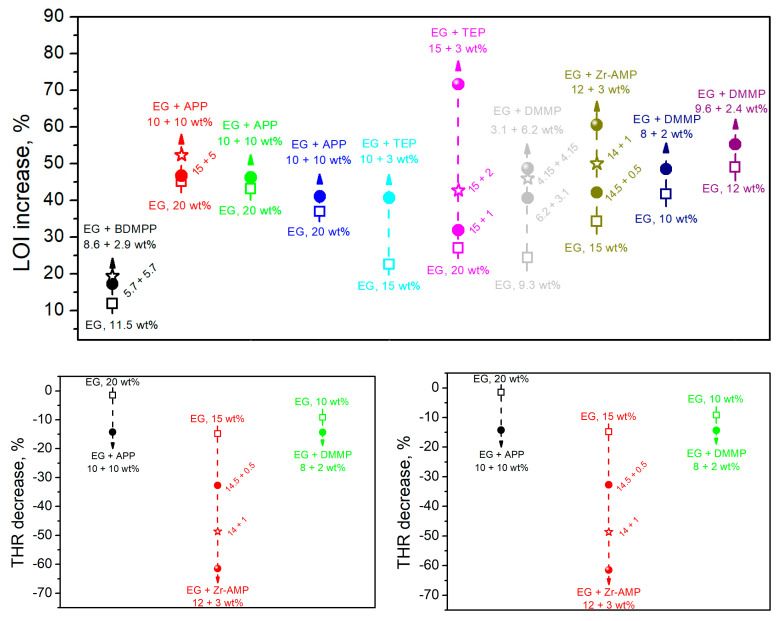
The impact of expandable graphite (EG)–phosphorous flame retardants (FRs) synergism on the limiting oxygen index (LOI) increase (based on the literature data: 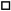 [59], 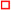 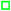 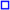 [53], 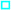 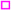 [33], 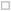 [60], 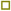 [61], and 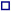 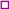 [55]), peak heat release rate (pHRR) decrease (based on the literature data: 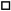 [53], 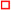 [61], and 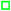 [55]), and total heat release (THR) decrease (based on the literature data: 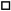 [53], 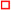 [61], and 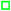 [55]) for polyurethane (PU) foams. The following abbreviations were used: APP—ammonium polyphosphate, BDMPP—bis( [dimethoxyphosphoryl] methyl) phenyl phosphate, DMMP—dimethyl methyl phosphonate, EG—expandable graphite, TEP—triethylphosphate, and Zr-AMP—nano zirconium amino-tris-(methylenephosphonate).

Herein, in the presented work, we investigated the impact of FR systems consisting of EG, organophosphorous FRs, and surface-modified nanoclays on the synergistic flammability reduction in flexible PU composite foams containing waste-based filler ground tire rubber (GTR) originating from the mechanical recycling of post-consumer car tires. Along with our two recently published papers [50,51], it is part of a series of works dealing with the flammability reduction of such materials. Before, this issue was investigated only in one work by Ryszkowska et al. [52], who, did not report the results of the cone calorimeter analysis. Nevertheless, they pointed to the high efficiency of a combination of expandable graphite and organophosphorus FRs, which was also explored in the presented work. The EG-phosphorous FR synergism was repeatedly reported by multiple research groups, summarized in Figure 1. Notably, all of these cases (presented in Figure 1) assumed the reduction of the EG loading at the expense of phosphorous FRs. When phosphorous FRs were introduced additionally with the constant loading of EG, the enhancement of flame retardancy was even more significant, yielding LOI values often exceeding 30%, peak heat release rate (pHRR) values below 100 kW/m^2^, or total smoke release (TSR) below 300 m^2^/m^2^ [30,33,53,54,55].

As nanoclays were repeatedly reported to show very beneficial impacts on the PU foams’ flammability reduction [56,57,58], the presented work aimed to examine their impact on the efficiency of hybrid FR systems consisting of a combination of EG with organophoshorous FRs. The reported “labyrinth effect” limiting the heat and mass transfer should provide auspicious benefits to the formation of a protective char layer.

## 2. Materials and Methods

### 2.1. Materials

**Figure 2:**
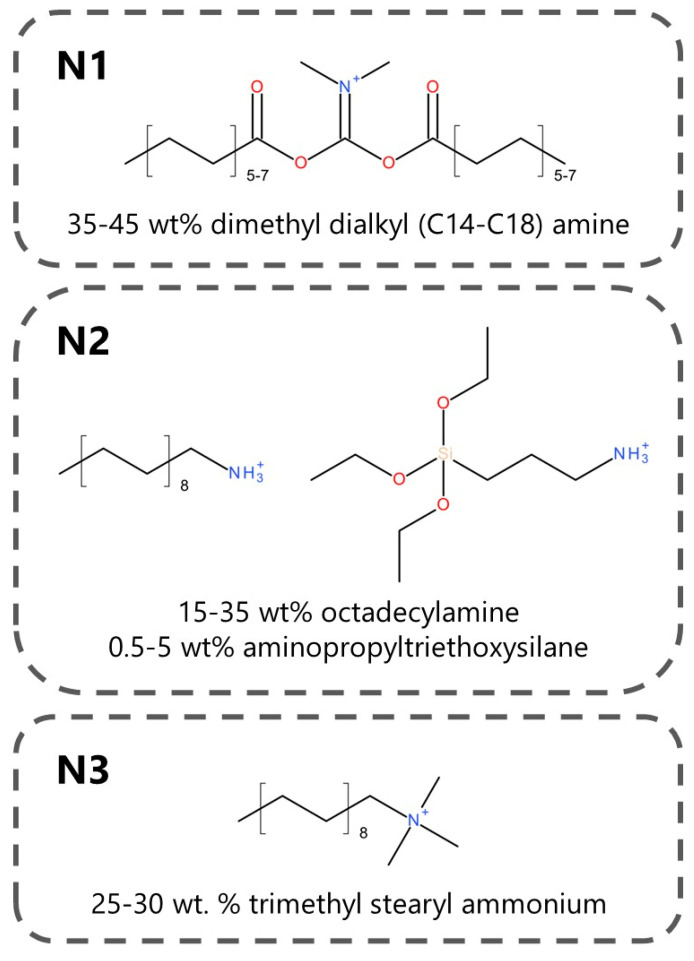
Chemical structures of surface modifiers of applied nanoclays.

The materials applied in the presented study are listed in Table 1. Moreover, Figure 2 presents the chemical structure of the surface modifiers of applied nanoclays.

### 2.2. Preparation of Composite Foams

Composite PU/GTR foams were prepared using a single-step method with an isocyanate index of 1:1. The GTR filler in the amount of 5 wt% was mixed with the polyols at 1000 rpm for 60 s to guarantee its proper distribution. Afterward, other components, except isocyanate, were added, including 20 wt% of FRs and 2 wt% of nanoclay, and mixing continued for another 30 s. Then, isocyanate was added, and the whole system was mixed for 10 s at 1800 rpm and poured into a closed aluminum mold with dimensions of 200 mm × 100 mm × 40 mm. After demolding, the samples were conditioned at room temperature for 24 h. Foams were coded A_X_B_Y_N_Z_, where A and B are FR abbreviations, X and Y point to their contents, N means nanoclay, and Z stands for the type of nanoclay.

### 2.3. Characterization

Fire behavior measurements of flexible PU/GTR composite foams were carried out using an iCone classic cone calorimeter from Fire Testing Technology (West Sussex, United Kingdom) as described by the ISO 5660 standard [62]. Samples with dimensions of 100 mm × 100 mm × 40 mm were subjected, in a horizontal position and in the presence of a spark from an igniter that initiated burning, to a heat flux of 50 kW/m^2^ produced by a conical electric radiant heater, and changes in the oxygen concentration of the combustion gases were measured. From the changes in oxygen concentration, the intensity of heat release was determined. An optical system with a silicon photodiode and a helium–neon laser delivered a survey of smoke. The parameters obtained during cone calorimetry analysis were applied to calculate the fire performance index (FPI) [63] and flame retardancy index (FRI) [64] of PU/GTR composite foams according to the following equations (1 and 2):

FPI = TTI/pHRR,(1)

FRI = (THR × pHRR/TTI)_Neat material_/(THR × pHRR/TTI)_Flame-retarded material_, (2)

where THR is the total heat released, MJ/m^2^; pHRR is the peak heat release rate, kW/m^2^; and TTI is the time to ignition, s. To evaluate the impact of applied FRs on an FRI, a sample containing 5 wt% of GTR without FRs was considered neat material.

## 3. Results and Discussion

Cone calorimetry is a very versatile method that enables the determination of numerous parameters describing the combustion process, which, for the prepared PU/GTR composite foams, have been summarized in Table 2. Among the analyzed parameters were time to ignition (TTI), peak and mean heat release rate (pHRR and mHRR), the maximum average rate of heat emission (MARHE), total heat release (THR), peak effective heat of combustion (EHC), residue at flameout, total smoke release (TSR), mean specific extinction area (SEA), mean carbon monoxide yield (COY), and mean carbon dioxide yield (CO_2_Y). Moreover, Table 2 provides values of composites’ FPI and FRI parameters.

Considering TTI, which somehow quantifies the ignitability of material, no significant differences between analyzed samples were noted. Although the elongation of TTI can be desired, as it may facilitate avoiding a fire situation, significant TTI enhancement in the case of PU foams is an enormous challenge. Due to the complexity of the PU structure, predicting, modeling, and inhibiting ignition are very challenging, as they typically follow a multi-step mechanism strongly influenced by the external conditions [65]. Therefore, researchers typically focus more on taming the actual fire and reducing its threats.

The HRR is often considered the most critical parameter of combustion, as it quantifies the size of the fire and its potential to spread. Higher values increase the potential ignition of the surrounding and its elements. Cone calorimetry provides information about the peak and mean values of HRR. Peak values describe the most critical moment of fire, which may often determine if other surrounding elements, typically characterized by lower flammability than analyzed materials, can be ignited [66,67]. On the other hand, mHRR provides information on the whole fire course, which often lasts dozens of minutes. The most significant differences in pHRR and mHRR among the analyzed samples were attributed to the EG content. Following the literature data, the introduction of EG enables a significant, almost 70% drop in the pHRR of PU foams [59,61,68]. In the presented case, increasing its content from 10 to 15 wt% at the expense of organophosphorus FRs reduced HRR values, due to the efficient creation of a carbonaceous layer during thermally induced volumetric expansion [33]. Its protective effect is attributed to the limited heat and mass transfers, which inhibit the fire spread by inhibiting the flashover phenomenon [69]. When combined with phosphorous FRs, EG generates structure additionally covered with a highly viscous layer yielded from phosphorous-catalyzed charring [30]. As a result, the char layer is significantly stronger and does not crack during combustion yielding in enhanced heat release, limiting the protective impact. Considering the type of applied nanoclay, no straightforward impact has been noted. However, in the case of higher EG loadings, the N2 type was more efficient in reducing the mean HRR value, which may suggest a more efficient char layer formation, potentially associated with the presence of silane and the formation of a denser and stronger structure, as indicated by the literature data [70,71].

The MARHE parameter enables fire spread evaluation and is often applied for regulatory purposes [72]. Nevertheless, as it aims to describe the complex phenomenon with one parameter, it often oversimplifies the conclusions or even misleads [73]. Therefore, MARHE-based conclusions are true only for a specific fire scenario, which is designed by the applied cone calorimeter test parameters [74]. In the presented case, MARHE followed a similar trend to pHRR and mHRR values and was most significantly affected by the increasing EG content, similar to the THR values. Such findings, once again, point to the high efficiency of EG in reducing PUs’ flammability.

On the other hand, such a trend was not noted for the EHC, which quantifies the efficiency of a flame retardant in terms of flame inhibition action by activity in the gas phase [75]. Considering all of the analyzed FRs’ formulations, they contained high shares of EG, whose mode of action in the gas phase is only related to the dilution with a non-flammable gas mixture of water vapor, sulfur, and carbon dioxides. The increase in the organophosphorus FRs’ content did not affect the EHC values, even though such compounds typically actively inhibit fire in the gas phase [37]. It can be concluded that the beneficial impact of phosphorous FRs is counterbalanced by the reduction in EG content and the limited dilution of the gas phase. Nevertheless, compared to our previous work [76], where we reported the use of F6_5_EG_15_ and F6_10_EG_10_ compositions without nanoclays to reduce the flammability of PU/GTR composite foams, the EHC values were 68–71% lower. Such a decrease indicates the high efficiency of nanoclays in terms of creating a “labyrinth effect”, inhibiting heat and mass transfers to the gas phase. A similar synergistic effect was noted by Thi et al. [77], who attributed it to the limited permeability of oxygen into the material and reduced evaporation of degradation products. Nanoclays, due to their particle size enhancing their homogenous distribution, may act in the condensed phase, protecting the not-yet combusting part of the material by limited heat transfer, but it may also contribute to the strengthening of the EG-originated char layer.

The high efficiency of nanoclays in forming a protective layer was also evidenced by the significantly higher values of char residue than reported in our previous works [50,51,76], which were also boosted by the high EG loading. Moreover, values of TSR and SEA were significantly lower than for the aforementioned F6_5_EG_15_ and F6_10_EG_10_ compositions, which confirmed the benefits of the “labyrinth effect” limiting gas permeability. The most significant difference was noted for the higher EG loading. Without nanoclays, TSR and SEA values were 1181 m^2^/m^2^ and 214 m^2^/kg, respectively, while the presented work reported noticeably lower values.

All of these effects confirm the high efficiency of the protective char layer generated during combustion, as well as the synergism between EG, organophosphorus FRs, and nanoclays. It is a common observation in the case of PU foams that multi-component systems are by far more efficient than even the best single FRs [48,78,79,80]. It is associated with the characteristics of PU foams and their cellular structure, which is highly sensitive to formulation changes affecting cells’ nucleation and coalescence, as well as the strength of the PU scaffold. Single FRs are often very efficient but at high loadings, which disrupts the cellular structure and results in the deterioration of insulation or the damping performance, which is often critical for PU foams’ industrial applications.

The synergism between different FRs was greatly described by Okrasa et al. [81]. They analyzed the impacts of different organophosphorus FRs on the combustion parameters of PU foams determined by cone calorimetry. For their sole application, all of the analyzed parameters deteriorated, which was attributed to the inefficient formation of a protective char layer. On the other hand, a combination with EG enabled the formation of a thicker layer, which provided an appropriate temperature gradient and effectively inhibited heat and mass transfers between PUF and the flaming zone, yielding pHRR, MARHE, THR, or TSR values reduced by over 50%. The synergism between EG and organophosphorus FRs is based on the release of phosphoric and polyphosphoric acids, which dehydrogenate the polymer and yield a highly viscous liquid film, strengthening the char layer [30]. Considering the mode of action of nanoclays related to the formation of a protective layer and the “labyrinth effect,” they potentially provided additional input to the discussed synergism, which enabled the significant enhancement of fire performance, compared to our previous works.

Finally, Table 2 reported values of FPI and FRI, two parameters that aimed to quantify the efficiency of FRs or their combinations in flammability reduction. For the calculation of FRI, the THR, pHRR, and TTI values for neat material (PU/GTR composite without FRs) were 166.5 MJ/m^2^, 461.1 kW/m^2^, and 8 s, respectively. The values presented for FPI were relatively low at the level of unmodified thermoplastic polymers. According to Vahabi et al. [64], an FRI value exceeding 10 points to excellent flame-retardant performance. However, initially, it was developed for thermoplastic polymers, characterized by different combustion behaviors, due to the lack of cells filled with gas, so the thresholds for foams could be set at different values.

**Figure 3:**
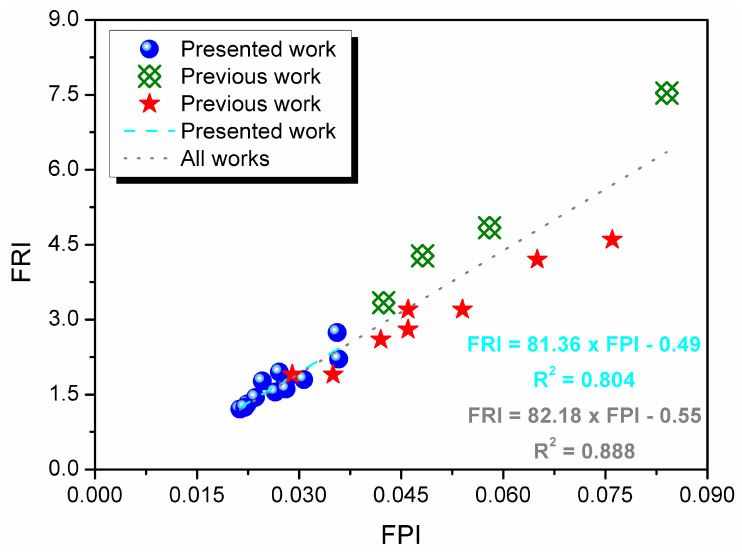
The correlation between FPI and FRI values calculated for the analyzed composites, as well as for the flexible foamed PU/GTR composites reported in our previous works: 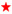 [50], Kliknij lub naciśnij tutaj, aby wprowadzić tekst. 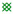 [51].

The FPI is considered a reasonable first-order indicator of propensity to flashover, while FRI was developed to assess flame retardancy more comprehensively [63,82]. The FPI provides value, which quantifies the performance of the whole material. At the same time, FRI measures the efficiency of applied FRs or their combinations, which noticeably facilitates the comparison between different polymer matrices and matches their needs with the mode of action of particular additives. Nevertheless, in the presented case, the correlation between FPI and FRI was relatively strong, as indicated by Figure 3. Moreover, in the analysis of the FPI–FRI relationship, we included results reported in our previous works on the PU/GTR composite foams modified with various FRs (including, applied here, liquid organophosphorus FRs and EG but also ammonium polyphosphate, melamine cyanurate, and melamine polyphosphate), which have been used in different manners (as simple additives for foams or GTR modifiers). A relatively good correlation coefficient points to a good efficiency of the FPI parameter in describing the flammability reduction of polymer composites; however, this marks the fact that all of the analyzed materials were based on a similar PU/GTR composite matrix.

## 4. Conclusions

The results reported in the presented work confirmed that reducing the flammability of PU foams is quite a challenge, especially when introducing highly flammable fillers like GTR. It aimed to evaluate the input of nanoclays to the synergistic flammability reduction in flexible PU/GTR composite foams. Despite the lack of a clear correlation between the surface modifications of applied nanoclays, the data obtained from the cone calorimeter and its comparison with our previous works pointed to the high efficiency of applied FRs’ combinations. The effect was powerful when nanoclays were combined with the higher loadings of EG, which enhanced the formation of the protective char layer. Based on the reported results, further work should evaluate different EG:nanoclay ratios and additional treatments aimed at improving the dispersion of solid FR particles in the PU matrix, which may further enhance the formation of the carbonaceous layer.

## Figures and Tables

**Table 1:** List of compounds used during the manufacturing of analyzed composites.

Component	Trade Name	Producer	Component	Trade Name	Producer
Polyols	Rokopol^®^ F3000	PCC Group (Brzeg Dolny, Poland)	Blowing agent	Distilled water	---
	Rokopol^®^V700	PCC Group (Brzeg Dolny, Poland)	Ground tire rubber (GTR)	---	Recykl S.A. (Śrem, Poland)
	Glycerol	Sigma Aldrich (Poznań, Poland)	Flame retardants	Roflam F6	PCC Group (Brzeg Dolny, Poland)
Diisocyanate	SPECFLEX NF 434	M. B. Market Ltd. (Baniocha, Poland)		Roflam B7	PCC Group (Brzeg Dolny, Poland)
Catalysts	PC CAT^®^ TKA30	Performance Chemicals (Belvedere, UK)		Expandable graphite (EG)	Nordmann, Rassmann, GmbH (Hamburg, Germany)
	Dabco33LV	Air Products (Allentown, PA, USA)	Nanoclays	Nanomer^®^ I.44P (N1)	Nanocor, Inc. (Arlington Heights, IL, USA)
	Dibutyltin dilaurate	Sigma Aldrich (Poznań, Poland)		Nanomer^®^ I.31PS (N2)	Nanocor, Inc. (Arlington Heights, IL, USA)
				Nanomer^®^ I.28E (N3)	Nanocor, Inc. (Arlington Heights, IL, USA)

**Table 2:** Results of cone calorimeter evaluation of prepared PU/GTR composite foams. Values in parentheses are standard deviations.

Sample	TTI, s	pHRR, kW/m^2^	mHRR, kW/m^2^	MARHE, kW/m^2^	THR, MJ/m^2^	EHC, MJ/kg	Residue, wt%	TSR, m^2^/m^2^	SEA, m^2^/kg	COY, kg/kg	CO_2_Y, kg/kg	FPI	FRI
F6_5_EG_15_N_1_	5 (0)	141 (39)	74 (11)	141 (34)	125 (16)	23 (2)	43.9 (2.0)	306 (10)	56 (5)	0.021 (0.008)	1.55 (0.11)	0.0356	2.74
F6_5_EG_15_N_2_	4 (0)	163 (10)	72 (1)	154 (15)	133 (0)	24 (0)	41.4 (0.2)	351 (32)	51 (6)	0.043 (0.012)	1.58 (0.04)	0.0246	1.77
F6_5_EG_15_N_3_	5 (0)	139 (1)	94 (2)	126 (22)	156 (7)	26 (1)	37.1 (1.6)	608 (25)	102 (2)	0.037 (0.010)	1.63 (0.01)	0.0358	2.21
F6_10_EG_10_N_1_	5 (0)	189 (19)	128 (1)	170 (11)	164 (3)	23 (1)	25.8 (0.1)	1965 (142)	276 (26)	0.029 (0.006)	1.53 (0.01)	0.0265	1.55
F6_10_EG_10_N_2_	4 (0)	179 (41)	93 (1)	162 (32)	164 (2)	23 (0)	27.0 (1.0)	2016 (125)	286 (15)	0.016 (0.010)	1.53 (0.00)	0.0224	1.31
F6_10_EG_10_N_3_	4 (1)	165 (23)	96 (6)	157 (2)	169 (4)	24 (0)	27.4 (3.7)	1651 (90)	232 (19)	0.032 (0.010)	1.54 (0.01)	0.0213	1.21
B7_5_EG_15_N_1_	4 (1)	125 (18)	84 (6)	116 (22)	155 (6)	25 (0)	35.5 (2.8)	768 (7)	122 (3)	0.029 (0.012)	1.60 (0.05)	0.0280	1.74
B7_5_EG_15_N_2_	4 (1)	129 (22)	71 (7)	129 (10)	133 (10)	23 (1)	40.7 (3.4)	482 (22)	81 (10)	0.022 (0.009)	1.56 (0.04)	0.0271	1.95
B7_5_EG_15_N_3_	4 (1)	169 (14)	90 (8)	143 (16)	158 (9)	25 (1)	35.5 (1.0)	897 (114)	137 (21)	0.047 (0.003)	1.59 (0.04)	0.0236	1.44
B7_10_EG_10_N_1_	5 (1)	178 (13)	93 (3)	175 (12)	168 (8)	24 (0)	28.3 (2.3)	1514 (91)	213 (32)	0.027 (0.011)	1.56 (0.02)	0.0281	1.61
B7_10_EG_10_N_2_	6 (2)	179 (15)	102 (2)	178 (16)	164 (2)	23 (1)	27.4 (0.5)	1829 (147)	259 (15)	0.024 (0.011)	1.58 (0.01)	0.0307	1.80
B7_10_EG_10_N_3_	4 (0)	182 (2)	106 (0)	171 (3)	171 (7)	25 (0)	29.8 (1.3)	1795 (85)	264 (5)	0.034 (0.007)	1.54 (0.01)	0.0220	1.24

## Data Availability

The original contributions presented in the study are included in the article, further inquiries can be directed to the corresponding author.
